# 2201. Antibiotic-Prescribing Practices for Ambulatory Patients with Urinary Tract Infections in the Emergency Department

**DOI:** 10.1093/ofid/ofad500.1823

**Published:** 2023-11-27

**Authors:** Naveera Khan, Sarah J Norman, Sheena Ramdeen

**Affiliations:** MedStar Health/Georgetown - Washington Hospital Center Program, Silver Spring, Maryland; MedStar Washington Hospital Center, Bethesda, Maryland; MedStar Washington Hospital Center, Bethesda, Maryland

## Abstract

**Background:**

Data on outpatient antibiotic stewardship for urinary tract infections (UTIs) are scarce. We describe the antibiotic-prescribing practices for ambulatory patients with UTI in the Emergency Department (ED) of a tertiary care center.

**Methods:**

Consecutive sampling was used to identify non-hospitalized adult ED patients between January 1st 2022 and May 1st  2022, who had a urine culture or urinalysis and a diagnosis of UTI, dysuria, hematuria, urinary frequency, flank pain, or abdominal pain per ICD-10 codes. Patients with an indwelling urinary catheter within 48 hours of presentation or a diagnosis of a concurrent non-genitourinary tract infection were excluded. Of these, 115 were randomly selected. Excess antibiotic days were defined as the difference between the prescribed days and the maximum days recommended by the Infectious Diseases Society of America for a regimen.

**Results:**

Women comprised 78.3% of the sample; mean age 45.1 +/- 19.5 years. There were no patients with a history of multidrug-resistant UTIs or ≥ 2 UTIs in the preceding 6 months. Uncomplicated cystitis (n=36) was treated with beta-lactams (55.6%), nitrofurantoin (41.7%) and trimethoprim-sulfamethoxazole (2.8%). Patients who received beta-lactams had a mean age of 56 +/- 23.6 years, with no documented rationale for using beta-lactams over other agents in any patient. The mean excess days of antibiotics for patients with uncomplicated cystitis was 0.94 days, most commonly with nitrofurantoin (mean excess days 1.8). There were 19 non-pregnant asymptomatic patients, 52.6% of these received antibiotics (median age 77.5 years) despite not meeting UTI diagnostic criteria. All patients with pyelonephritis were prescribed beta-lactams; mean duration 11.3 +/- 2.8 days. Of the 7 patients with negative urine cultures that resulted after ED discharge, none were contacted to stop antibiotics.

**Conclusion:**

Antibiotic choice and duration, antibiotic prescription for non-pregnant asymptomatic patients, and lack of antibiotic de-escalation are potential areas to target antibiotic stewardship for UTIs in the ED for non-hospitalized patients.

**Disclosures:**

**All Authors**: No reported disclosures

